# Early Markers of Atherosclerotic Disease in Individuals with Excess Weight and Dyslipidemia

**DOI:** 10.5935/abc.20160060

**Published:** 2016-06

**Authors:** Eduardo Menti, Denise Zaffari, Thais Galarraga, João Regis da Conceição e Lessa, Bruna Pontin, Lucia Campos Pellanda, Vera Lúcia Portal

**Affiliations:** Instituto de Cardiologia - Fundação Universitária de Cardiologia, Porto Alegre, RS - Brazil

**Keywords:** Atherosclerosis, Biomarkers, Endothelium, Obesity, Dyslipidemias

## Abstract

**Background:**

Excessive weight is a cardiovascular risk factor since it generates a chronic inflammatory process that aggravates the endothelial function.

**Objective:**

To evaluate the endothelial function in individuals with excess weight and mild dyslipidemia using brachial artery flow-mediated dilation (BAFMD), and the association of endothelial function with anthropometric and biochemical variables.

**Methods:**

Cross-sectional study that included 74 individuals and evaluated anthropometric variables (body mass index [BMI], waist-hip ratio [WHR], waist circumference [AC], and percentage of body fat [PBF]), biochemical (blood glucose, insulinemia, ultrasensitive C-reactive protein, fibrinogen, total cholesterol, HDL-cholesterol, triglycerides, and LDL-cholesterol) and endothelial function (BAFMD, evaluated by ultrasound). The statistical analysis was performed with SPSS, version 16.0. To study the association between the variables, we used chi-square, Student's t and Mann-Whitney tests, and Pearson's correlation. Logistic regression analyzed the independent influence of the factors. Values of p < 0.05 were considered significant.

**Results:**

The participants had a mean age of 50.8 years, and 57% were female. BMI, WC, WHR, and PBF showed no significant association with BAFMD. The male gender (p = 0.02) and higher serum levels of fibrinogen (p = 0.02) were significantly and independently associated with a BAFMD below 8%.

**Conclusions:**

In individuals with excess weight and mild untreated dyslipidemia, male gender and higher levels of fibrinogen were independently associated with worse BAFMD.

## Introduction

When endothelial cells are exposed to risk factors such as hypertension, smoking, insulin resistance, and obesity, they are stimulated to express adhesion molecules on their surface, recruiting several classes of leukocytes and promoting the initial signaling mechanisms for cellular changes and atheroma formation.^1-4^ Endothelial dysfunction may be detected even before the occurrence of obstructive atherosclerotic plaques.^5^ The amount of nitric oxide released by endothelial cells depends on the integrity of the endothelium and determines the degree of vasodilation.^6^ The most used method to estimate endothelial dysfunction is the evaluation of the brachial artery diameter before and after distal tissue ischemia (hyperemic reaction).^7^ This measurement has applications in population studies, but its individual application has not been established yet.^8-10^ Dilation values between 8 and 10% seem to be the best discriminators between normal and abnormal endothelial functions.^8,11^

Obesity and excessive weight are able to change the vascular endothelium function.^12,13^ There is growing recognition that obesity is characterized by a low degree of chronic and subclinical inflammation.^14,15^ The exact mechanisms that stimulate this sustained inflammation have not been elucidated yet but are highly relevant to the atherothrombotic process.^16,17^

It is, thus, crucial to identify variables that could predict the progression of the disease and the occurrence of clinically significant events in obese individuals. This study evaluated the occurrence of associations of anthropometric measures and metabolic and inflammatory markers with endothelial function assessed by brachial artery dilation in individuals with excess weight and mild untreated dyslipidemia. The objective was to identify the variable with a better ability to predict the occurrence of subclinical atherosclerosis and, consequently, more useful in the clinical follow-up of individuals with excess weight.

## Methods

This study is part of a research conducted at *Instituto de Cardiologia* involving individuals with excess weight and dyslipidemia. The sample was obtained by convenience, and the study of the endothelial function was performed in one in every four participants undergoing nutritional and anthropometric follow-up, in a total of 74 individuals.

### Inclusion criteria

The study included men and women aged 35-60 years, with dyslipidemia and excess weight, and without a history of clinically manifested cardiovascular disease. Dyslipidemia was considered present when the levels of at least one of the following biochemical parameters was abnormal: total cholesterol (TC) > 200 mg/dL, and/or triglycerides (TG) > 150 mg/dL, and/or HDL-cholesterol < 40 mg/dL in men and < 50 mg/dL in women. Excess weight was assessed with the body mass index (BMI), and the participants had BMI values between 25 and 35 kg/m^2^.

### Exclusion criteria

Exclusion criteria were the occurrence of neoplasms, infections, and liver, kidney and gastrointestinal disorders; levels of LDL-cholesterol > 160 mg/dL and TG > 400 mg/dL; pregnancy and lactation; alcohol consumption above four doses a day; use of estrogen, nonsteroidal anti-inflammatory, antiobesity agents, and vitamin supplementation; use of statins, fibrates, and other lipid-lowering medications; unexplained weight loss (greater than 2 kg) in the last 30 days.

### Ethical aspects

The study was approved by the Ethics Committee in Research (*Comitê de Ética em Pesquisa,* COEP) at *Fundação Universitária de Cardiologia*. All patients were informed about the study by reading and analyzing the free and informed consent form and agreed to participate. The research protocol did not interfere with any medical recommendation or prescription.

### Study protocol

The selected individuals answered a standardized questionnaire and their anthropometric measurements (BMI, waist circumference [WC], waist-hip ratio [WHR], and body fat percentage), metabolic profile (blood glucose, insulin, TC, HDL-cholesterol, and TG), and inflammatory profile (C-reactive protein [CRP] and fibrinogen) were analyzed. The endothelial function was assessed with brachial artery flow-mediated dilatation (BAFMD). The technique used in this study was that recommended by the American Society of Echocardiography and Society of Vascular Medicine and Biology, based on the percentage modification of the brachial artery diameter by reactive hyperemia^7^.

### Statistical analysis

The results are presented as mean ± standard deviation for continuous variables. WC, WHR, and BMI were treated as qualitative variables using cutoff points described in the literature for values considered abnormal. Values of WC and WHR were considered abnormal in men when above 102 cm and 0.9, respectively, and in women when above 88 cm and 0.85, respectively. Values of BMI between 25 and 30 kg/m^2^ were considered as overweight and those equal to or above 30 kg/m^2^ as obesity. The association of the variables was analyzed with the chi-square test for dichotomous variables, Student's *t* test for parametric continuous variables, and Mann-Whitney test for nonparametric continuous variables. Results of ultrasensitive CRP (usCRP) are presented as median since this is a variable with a non-Gaussian distribution. Differences were considered statistically significant for p values < 0.05. Additionally, logistic regression was conducted to assess the independent influence of factors significantly associated with the endothelial vasodilation response and Pearson's correlation test to estimate the degree of linear relationship between the serum level of fibrinogen and the percentage of dilation of the brachial artery. We used the statistical program SPSS, version 16.0 (SPSS Inc., Chicago, USA).

## Results

The participants had a mean age of 50.88 ± 6.14 years, and 57% were female. All individuals had excess weight with a mean BMI value of 28.82 ± 2.60 kg/m^2^ and some degree of dyslipidemia, with mean values of TC of 222.67 ± 34.24 mg/dL, HDL-cholesterol of 45.68 ± 14.83 mg/dL, LDL-cholesterol of 146.05 ± 32.02 mg/dL, and TG of 154.66 ± 79.37 mg/dL (Table 1). The WC was increased in 46.9% of the men and 75.0% of the women while the WHR was abnormal in 90.5% of the men and 38.1% of the women. The percentage of body fat varied between 14.81% and 36.14%, with a mean value of 23.19 ± 4.12%. Only eight individuals had body fat percentage values above those compatible with obesity (25% in men and 32% in women). The individuals were then subdivided into groups of overweight and obesity. According to this criterion, 29.7% of the sample was composed of obese individuals.

**Table 1:** Characteristics of the cohort

**Characteristic**	**n**	**Statistics**
Age (years)	74	50.88 ± 6.14
Female gender (%)	74	42 (57%)
Smokers (%)	74	11 (14.8%)
Body mass index (kg/m²)	74	28.82 ± 2.60
Waist circumference (cm)	74	M: 101.48 ± 7.25
		F: 95.90 ± 12.90
Waist/hip ratio	74	M: 0.93 ± 0.05
		F: 0.83 ± 0.06
Percentage of body fat (%)	74	M: 21.53 ± 3.28
		F: 24.45 ± 4.29
Insulin	74	10.57 ± 6.09
Blood glucose (mg/dL)	74	101.45 ± 29.45
Total cholesterol (mg/dL)	74	222.67 ± 34.24
HDL-cholesterol (mg/dL)	74	M: 39.52 ± 8.44
		F: 50.24 ± 16.73
LDL-cholesterol (mg/dL)	74	146.05 ± 32.02
Triglycerides (mg/dL)	74	154.66 ± 29.45
Fibrinogen (mg/dL)	74	266.00 ± 63.06
Ultrasensitive C-reactive protein (mg/L) *	74	0.29 ± 0.31

Data are presented as mean ± standard deviation and median *or value (percentage).

HDL-cholesterol: high-density cholesterol; LDL-cholesterol: low-density lipoprotein cholesterol; M: male; F: females.

The diameter of the brachial artery varied 7.80 ± 6.41% during the BAFMD when compared with its baseline value (Table 1). The median BAFMD value was 8%, which served as a cutoff point for a qualitative analysis between individuals with vasodilation responses above and below this value.

**Figure 1:**
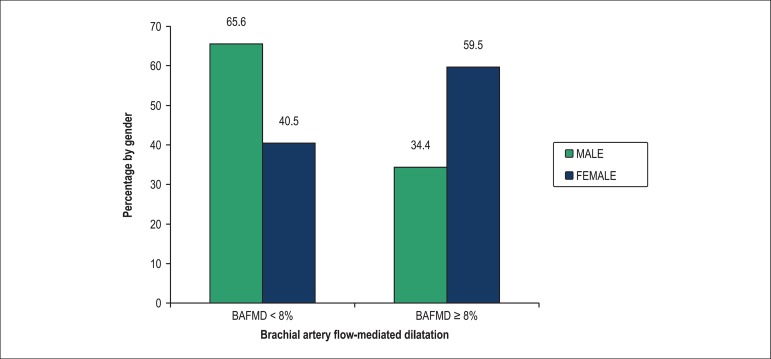
Association between gender and brachial artery flow-mediated dilatation. BAFMD: Brachial artery flow-mediated dilatation.

WC, WHR, and BMI, treated as qualitative variables, showed no association with the degree of vasodilation response treated as a continuous variable (verified by Student's *t* test) or qualitative variable (verified with the chi-square test, with a cutoff point of 8% for the BAFMD result) (Table 2). The male gender showed a significant association with a worse vasodilation response, *i.e*., men had more frequently BAFMD values below 8% (p = 0.03) (Figure 1).

**Table 2:** Association between anthropometric, metabolic and inflammatory variables with brachial artery flow-mediated dilatation

**Variable**	**BAFMD < 8%**	**BAFMD ≥ 8%**	**p**
Male gender	21	11	p = 0.03
BMI > 30 kg/m^(2†^	10	12	p = 0.09
Abnormal WC ^(†^ Men: > 102 cm; Women: > 88 cm	24	29	p = 0.83
Abnormal WHR ^(†^ Men: > 0.85; Women: > 0.90	21	19	p = 0.51
Percentage of body fat ^(‡^	23.04	23.34	p = 0.22
Insulin ^(‡^	9.60	11.63	p = 0.15
Blood glucose	99.60	103.00	p = 0.59
LDL-cholesterol ^(‡^	146.50	145.57	p = 0.90
HDL-cholesterol ^(‡^	42.63	49.00	p = 0.06
Triglycerides ^(‡^	167.11	141.14	p = 0.16
Fibrinogen ^(‡^	281.55	248.62	p = 0.02
UsCRP ^(*^	0.17	0.36	p = 0.14

* nonparametric variable, association verified with the Mann-Whitney test;

† association verified with the chi-square test;

‡ parametric variables, association verified with Student's t test.

BAFMD: brachial artery flow-mediated dilatation; BMI: body mass index; WC: waist circumference; WHR: waist/hip ratio; LDL-cholesterol: low-density lipoprotein cholesterol; HDL-cholesterol: high-density cholesterol; UsCRP: ultrasensitive C-reactive protein.

**Figure 2:**
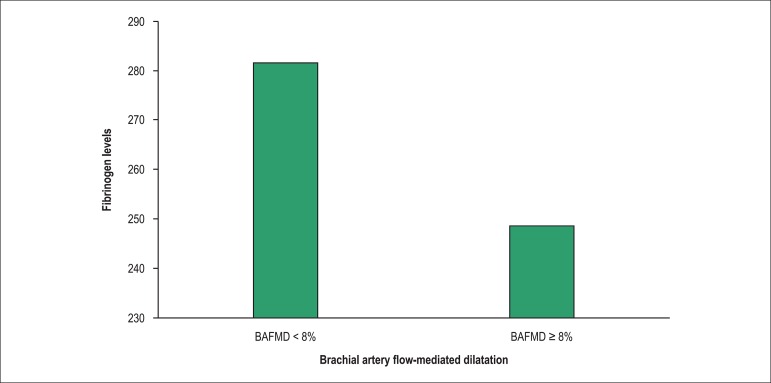
Association between fibrinogen levels and brachial artery flow-mediated dilatation. BAFMD: Brachial artery flow-mediated dilatation

**Figure 3:**
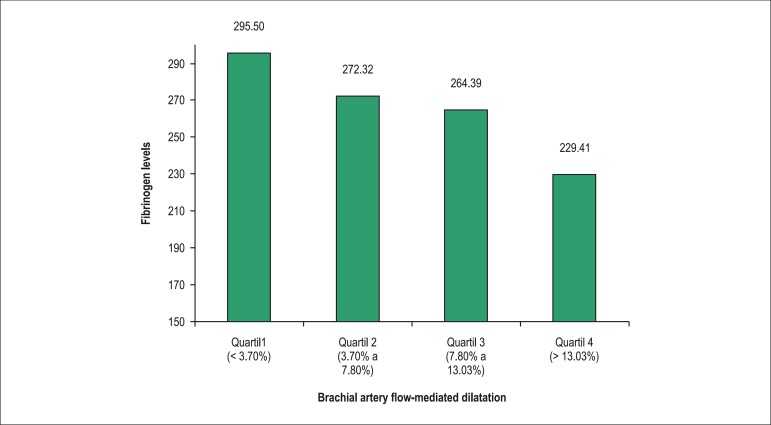
Distribution of serum fibrinogen levels by quartiles of brachial artery flow-mediated dilatation results.

The biochemical results of the metabolic parameters and inflammatory markers were treated as quantitative variables and their associations with the endothelial function were verified with Student's *t* test (Table 2). Fibrinogen was the only biochemical parameter significantly associated with the endothelial function (p = 0.02) (Figure 2). When this association was evaluated by quartiles of dilation, we observed that for dilatation values below 3.7%, the mean serum fibrinogen was of 295.50 ± 50.41 mg/dL, whereas for dilation values greater than 13.03%, the mean was 229.41 ± 48.95 mg/dL (Figure 3).

After we had observed the association of the male gender and serum fibrinogen level with worse brachial artery vasodilation response, we performed a logistic regression analysis to verify whether this would be an independent association. The results demonstrated that the associations between endothelial function with male gender and serum levels of fibrinogen remained significant. The male gender increased the chances of a worse vasodilation response by approximately three times (odds ratio [OR] 3.33; 95% confidence interval [CI] 1.19 - 9.28, p = 0.02), while an increase in 1 mg/dL in serum fibrinogen level increased this risk in 1% (OR 1.01, 95%CI 1.00 - 1.01, p = 0.02). Therefore, it would be expected that an increase of 100 mg/dL in serum fibrinogen level would increase in approximately two times the risk of a worse vasodilation brachial artery response.

The variables were additionally evaluated with Pearson's correlation test, and the correlation factor with the dilation of the brachial artery for fibrinogen was -0.31 (p = 0.008).

## Discussion

In a cohort of individuals with excess weight, mild dyslipidemia, and without clinically significant atherosclerotic disease, we found that the male gender and high levels of serum fibrinogen were associated with worse endothelial function determined by BAFMD. Our study suggests the relevance of measuring circulating fibrinogen as a marker of subclinical atherosclerosis in individuals with excess weight without manifested atherosclerotic disease.

The association of the male gender with worse endothelial function is aligned with clinical and epidemiological observations that the male gender is an important risk factor for atherosclerotic disease. By studying the influence of risk factors on endothelial function in asymptomatic individuals, different researchers have demonstrated an independent and significant association of the male gender with worse BAFMD.^18-20^ The inclusion of individuals with a mean age of 50 years in our study confirms this association, since at this age men have a higher cardiovascular risk than women.

Elevated fibrinogen levels are strongly associated with atherosclerotic disease. The ARIC (Atherosclerosis Risk in Communities) study has shown an increased risk of coronary disease with higher levels of fibrinogen, with a relative risk of 1.76.^21^ In the PROCAM (Prospective Cardiovascular Münster) study, the occurrence of death due to coronary disease and nonfatal infarction was greater among individuals with higher levels of fibrinogen. In that study, fibrinogen levels were better risk predictors than BMI and levels of LDL-cholesterol.^22^ In a meta-analysis that included 22 studies evaluating the association between serum concentration of fibrinogen and cardiovascular disease, the estimated risk of events in individuals with levels of fibrinogen in the highest tercile was two times greater than that in individuals with levels in the lowest tertile (OR 1.99, 95%CI 1.85 - 2.12).^23^ In children or adolescents with overweight or obesity, fibrinogen has also been associated with usCRP elevation and with theoccurrence of four or more cardiovascular risk factors^24^. In contrast, the association between fibrinogen and markers of early atherosclerosis has already been demonstrated in studies evaluating the carotid myointimal thickening and BAFMD. In a series of asymptomatic individuals, elevated fibrinogen levels were significantly related to increased myointimal thickening, independent of other potentially confounding variables.^25^ The same has been observed in another study that evaluated fibrinogen and usCRP as markers of subclinical carotid atherosclerosis.^26^

Similarly, greater myointimal carotid thickening, worse BAFMD, and higher concentrations of E-selectin and thrombomodulin have shown association with serum fibrinogen levels in obese children.^27^ Fibrinogen has also been described as more frequently increased in individuals with type 2 diabetes mellitus with metabolic syndrome than in those without metabolic syndrome. In addition, fibrinogen increases the risk of microvascular diseases, including diabetic retinopathy.^28^ A small study that has only evaluated the influence of fibrinogen in endothelium-dependent vasodilation has observed an inverse relationship between plasma levels of fibrinogen and degree of BAFMD.^29^ When individuals with manifested heart disease are considered, fibrinogen also appears as a marker of worse brachial artery vasodilation response.^30^

High serum levels of fibrinogen may promote vascular disease by increasing blood viscosity, stimulating fibrin formation, or increasing platelet-platelet interaction. Fibrinogen may also be simply a marker of vascular disease without contributing for its progression.^31^ The hepatic production of fibrinogen is regulated by cytokines whose concentrations increase in response to different inflammatory processes. In this context, excess weight has been associated with a higher production of inflammatory cytokines by the adipose tissue. This inflammatory status is due to a dysfunction in the interaction between adipocytes and tissue macrophages.^4,15,32^ CRP is also an acute phase inflammatory protein and its baseline levels are independent risk predictors of myocardial infarction and stroke, showing correlation with fibrinogen levels.^33,34^ Our study did not confirm an association between CRP and fibrinogen, which can be explained in part by the non-normal distribution of the CRP levels and the low levels detected in the serum. Similarly, the study lacked power to test the association between fibrinogen levels and degree of excess weight. This relationship has already been demonstrated in previous studies focusing on WC,^35^ body fat,^36^ BMI, and WHR.^37^ The narrow range of variation of the anthropometric parameters in our cohort seems to have influenced the lack of association of the adiposity measurements with endothelium-dependent vasodilation.

Obese individuals have a low-degree chronic inflammatory condition that manifests with worse flow-mediated vasodilation response.^38,39^ A relationship has already been demonstrated between markers of prothrombotic status, like fibrinogen and prothrombin activity, with the degree of visceral adiposity and other cardiovascular risk factors.^40^

Weight reduction is able to revert the deleterious effect of excessive weight on endothelial function through mechanisms not yet fully known.^41-43^ These observations about fibrinogen levels in obese individuals bring an additional element to the final consideration that fibrinogen is intimately related to subclinical atherosclerotic disease in individuals with excess weight.^44^

### Study limitations

The results of this study suggest an association between male gender and fibrinogen levels with endothelial function in individuals with excess weight and dyslipidemia. However, since this was a cross-sectional study, it is unable to determine a cause-effect relationship between these variables.

The verification of the association between inflammatory markers and degrees of excess weight, as well as between the degrees of excess weight and endothelial dysfunction may have been compromised by the uniformity of the degrees of adiposity and the sample size.

## Conclusion

The results of this study suggest that fibrinogen is associated with subclinical atherosclerosis in individuals with excess weight. New studies should clarify this association and establish the benefit of including fibrinogen as a marker in clinical practice to evaluate this group of patients.
